# Considering social determinants of health in the relationship between physical activity and exercise engagement and cognitive impairment among persons with epilepsy

**DOI:** 10.3389/fresc.2022.923856

**Published:** 2022-07-26

**Authors:** Barbara Hansen, Jane B. Allendorfer

**Affiliations:** ^1^Division of Preventive Medicine, University of Alabama Heersink School of Medicine, Birmingham, AL, United States; ^2^Departments of Neurology and Neurobiology, University of Alabama Heersink School of Medicine, Birmingham, AL, United States

**Keywords:** epilepsy, cognitive impairment, physical activity, exercise, quality of life, social determinants of health, participation, access

## Abstract

Many persons with epilepsy (PWE) are not as active or physically fit as compared to the general population. This lack of engagement in physical activity has been attributed to a number of factors, few of which take into consideration the social determinants of health (SDH). In this perspective, we highlight how SDH are considered in explaining lower levels of physical activity engagement among PWE, particularly for those experiencing cognitive impairment. We also discuss how these data can be applied in research to yield a greater impact on the quality of life among PWE. Consideration of SDH allows for increased understanding of how cognition can be both a determinant of physical activity and an outcome of environments conducive to physical activity in PWE.

## Introduction

Exercise and physical activity play important roles in overall health and well-being; however, not everyone has equal opportunity when it comes to participation (1), particularly persons with epilepsy (PWE) (2). Epilepsy, characterized by seizure activity and the predisposition to recurrent unprovoked seizures (3), is second only to migraine headaches among the most common neurological disorders in the world, affecting ~50 million individuals (4).

In addition, nearly half of PWE experience at least one domain of cognitive impairment (5), which are typically attributed to type of seizures (6), use of anti-seizure drugs (7), and/or seizure frequency (6, 8). Evidence further suggests that PWE who also have cognitive deficits report a pronounced decrease in quality of life and limitations in their participation in daily activities including school, work, and exercise (9, 10). With this perspective, we aim to examine factors that influence participation and engagement in physical activity and exercise among PWE with cognitive impairment, and how resources play a role in access to healthy activities.

## Epilepsy incidence and prevalence in the U.S.

An estimated three million adults and 470,000 children reported having active epilepsy (i.e., ≥1 seizure in previous 12 months) in the U.S. (11, 12). An additional 87,000 children are reported to have new-onset epilepsy each year in the U.S. and prevalence varies by age, gender, and insurance type; steep increases occur in the first 3 years of life, cases are more likely to be males, and much more likely to have Medicaid than private insurance (13), The most recent National Health Interview Survey, noted that, among PWE, the vast majority of children received care from an epilepsy specialist, while in adults with active epilepsy, 53% reported treatment from both a neurologist and general doctor, 9% saw a neurologist or epilepsy specialist exclusively, and 27% sought epilepsy care only from a general doctor; unfortunately, 11% reported not seeing a general physician or neurologist for their condition (14).

Epilepsy and cognitive impairments often go hand-in-hand, having a devastating impact on PWE's quality of life. As Lenck-Santini and Scott found in a review of literature dating back to the 1980's, a common theme emerges with regard to information processing and memory, type of seizure, age of onset of epilepsy, length of time since diagnosis, treatment-resistant epilepsy vs. seizures well-controlled with medication, and co-occurring encephalopathies (15). Considering the significant variations in incidence, prevalence, and outcomes in epilepsy, a PWE's social characteristics should be examined to assess what, if any, impact they may have on outcomes. access to healthy activities.

## Social determinants of health

Social determinants of health (SDH) are social conditions related to the myriad of environments into which people are born, grow, and live that are shaped by the distribution of resources, which, in turn, affect health outcomes either positively or negatively and are largely responsible for inequalities in health outcomes (16). Over the past 30 years, since Whitehead and Dahlgren's work, researchers have been encouraged to examine associations between SDH and health outcomes in epidemiological studies of chronic conditions and to highlight disparities when reporting results (17). There are a number of SDH models in use, each with its own conception of which mechanisms drive inequalities; however, all models are rooted in the premise that socioeconomic inequalities drive health inequalities (18). An important consideration is that each disorder and global region have unique societal factors that should be accounted for when determining the paths of causality (18). For example, to address SDH in the field of disability, the World Health Organization established the International Classification of Functioning, Disability and Health (ICF), a system with a stated goal of studying health outcomes, determinants, and changes in health status for disabling conditions, such as epilepsy, in an effort to allow for comparison across countries and establishing a common language for persons involved in disability care and research (19). Within this framework, SDH would fall into the “environmental factors” category, which examines the physical, social, and attitudinal environments and whether these factors are barriers or facilitators to functioning.

## SDH and epilepsy

More than five million new patients are diagnosed with epilepsy each year and incidence and mortality vary widely between countries and regions (20). For instance, PWE residing in low-and-middle-income countries have a higher mortality rate, likely attributable to lack of access to medical facilities and anti seizure medications, as well as injuries sustained during seizures (21). Male sex and low socioeconomic status are also positively correlated to incidence of epilepsy (22, 23). Further, there is evidence that children's socioeconomic status is associated with time to surgery and surgical outcome, regardless of insurance status; children whose parents are in the lowest income bracket had significantly longer time to surgery and lower odds of achieving improved seizure frequency than those in the highest income levels (24). In other words, SDH, play a large role in epilepsy outcomes. Specific to the study of epilepsy, we think it is appropriate to further examine SDH through the lens of the SDH-Epilepsy conceptual framework, which incorporates the environmental factors in the ICF and expands the structural and intermediary determinants to include other factors, such as the political context, cultural and societal values, social capital, residential segregation, specific living conditions, and proximity to resources (25). Adoption of a more comprehensive list of SDH data collected in epilepsy research can provide valuable information about resources and social supports PWE have and insight into possible adherence issues with recommended medical and behavioral interventions.

## Cognitive impairment and physical activity and exercise in epilepsy

Overall, 26% of PWE also have a co-occurring cognitive impairment (26). This high prevalence has spurred the implementation of both invasive and non-invasive strategies to ameliorate the physical and psychological repercussions of epilepsy and seizures with mixed results (5, 27). Evidence supports that physical activity and exercise are strategies associated with improvement of cognitive functioning in general (28) and may be protective against age-related decline (29). However, data are limited for investigations of physical activity and exercise among PWE. Overall, studies have shown that increased physical activity and exercise in PWE are associated with improvement or no change in seizure control and improvements in health-related quality of life and mood symptoms (30). With respect to cognitive functioning, the few exercise intervention studies we identified, particularly using combined endurance and resistance training, consistently showed that exercise is associated with improvements in cognitive domains, including verbal memory, attention, and executive function (10, 31, 32). Another consistent finding is that PWE are less likely to engage in regular leisure time physical activity than their counterparts without epilepsy (30, 33–36), despite the potential and known cognitive and other benefits of exercise. Fear of provoking a seizure, physical limitations, lack of confidence in performing the exercise activity, and a lack of knowledge among sports instructors are just some of the factors influencing decreased engagement of PWE in physical activity and exercise (37–40).

In 2016, the International League Against Epilepsy Task Force on Sports and Epilepsy published a special report providing guidance regarding participation in sports activities, with relatively few limitations for seizure-free PWE (39). The report categorized sports activities by level of risk of injury or death if a seizure occurs during the event, with low/no additional risk activities including dancing, golf, racquet sports, collective contact sports like judo or wrestling, and collective ground sports like baseball, basketball, field hockey, football or volleyball. Recent data have shown that a 6-month program of moderate intensity cardiovascular exercise performed at home using an ergometric bicycle for 30 min/day for 5 days/week significantly improved cardiovascular fitness without effecting seizure frequency of adults with drug-resistant epilepsy (41). Two other recent studies in adults with epilepsy showed health and cognitive benefits with a supervised exercise program that combined endurance and resistance training for either three 60-min sessions/week for 6 weeks (31) or two 60-min sessions/week for 12 weeks that also included stretching exercises (32). Endurance training was performed with either a stationary bicycle (31) or treadmill (32) with target intensities set to moderate-vigorous levels. Resistance training for both studies employed free weights and/or exercise machines and focused on strengthening the major muscle groups using a protocol that progresses to performing 3 sets of 8–12 repetitions of maximal intensity for each strength exercise including chest press, leg press, and seated row (31, 32). The most recent exercise intervention study in children with epilepsy 8–12 years old showed neurocognitive and psychological benefits with a 5-week supervised program consisting of two 3-h sessions/week that included 2 different team activities each for 90 min (e.g., basketball, soccer, badminton, jump rope, line dance and table tennis), followed by a 30-week home-based exercise program (42). The home-based program consisted of 20–30 min of aerobic exercise and a goal of walking more than 7,000 steps/day, as well as performing body weight resistance exercises like push-ups and sit-ups (42). While PWE, particularly if not seizure-free, are recommended to be cautious regarding participation in certain types of exercise or sports due to potential concerns of injury (39), recent trends are showing that PWE can safely engage in both resistance and endurance exercises of moderate to vigourous intensities, specially with expert supervision. Home-based exercise programs for children and adults with epilepsy are also feasible and show significant benefits, and may increase overall accesibility of exercise for PWE. Together, these studies provide recent evidence regarding exercise modalities that are well-tolerated and show beneficial effects in PWE, which will hopefully encourage increased physical activity and exercise engagement.

## Context of SDH in physical activity and exercise among PWE

Although research over the past 10 years has informed us of social barriers to achieving optimal health generally among PWE (25, 43–45), contextual factors such as insufficient insurance, housing insecurity, employment status, nutrition, and health literacy are important to consider (25). Further, because PWE with co-occurring cognitive impairment are more likely to be disabled (46), the SDH of the patient's caregivers should be taken into consideration, as the levels of financial, physical, and emotional burden can be immense (47, 48). Regardless of disability status, these SDH can significantly impact a person's access to physical activity, particularly for those of minority race or ethnicity. For example, locale-specific distal SDH, including residential income levels (49), racial segregation (50, 51), living conditions (52, 53), neighborhood social cohesion (54–56), walkability (57, 58), and proximity to parks (59), can all play a role in how access to physical activity is distributed within a population. While Ablah et al. consider that the lower income levels of their study participants may have influenced adherence to structured physical activities (38), the vast majority of SDH, with respect to their abilities to affect the physical activity levels and exercise participation and engagement of PWE, have not been examined as of yet. In order to determine whether cognition is a determinant of physical activity or an outcome of environments conducive to physical activity, additional studies should be undertaken.

Because access to physical activity and exercise can be constrained by PWE and their families' SDH, examining previous findings that link SDH to cognitive outcomes among vulnerable populations can shed light on patient activities. Poverty is an excellent predictor of negative outcomes; lower social class at birth is associated with prenatal and early childhood nutritional deficiencies, which are, in turn, linked to developmental lags and lower educational achievement (60). Maternal hardship and unsafe living environments have profound consequences on children's health (61, 62) and, unsurprisingly, these physical and social environments are concentrated in under-resourced regions throughout the world (63). When race and ethnicity are included as factors, researchers have found that, while socioeconomic position may explain the increased magnitude of negative health outcomes (64), racism allows these disparities to endure (65). Findings indicate that the cumulative effects of stressors from racism and low socioeconomic status can follow persons of color throughout their life course (66).

## Conclusion

The lessons we have learned over the past 30 years of research into disproportionate outcomes due to SDH can be applied to PWE. As we look further into the benefits of physical activity and exercise on cognitive impairment in PWE, we should expand our understanding of SDH variables and consider the factors that are unique to our populations of interest so we may better address the social conditions contributing to disparate access to physical activity and exercise. Narrowing our research focus to those with additional barriers to physical activity, such as those with cognitive impairments co-occurring with epilepsy, can inform physical activity and exercise intervention strategies for this population and their families.

## Data availability statement

The original contributions presented in the study are included in the article/supplementary material, further inquiries can be directed to the corresponding author/s.

## Author contributions

BH and JBA conceived the theme. BH developed the manuscript, revised, and finalized the manuscript. JBA contributed to the manuscript development and organization, reviewed and edited the manuscript, and finalized the manuscript. Both authors approved the manuscript for submission.

## Conflict of interest

The authors declare that the research was conducted in the absence of any commercial or financial relationships that could be construed as a potential conflict of interest.

## Publisher's note

All claims expressed in this article are solely those of the authors and do not necessarily represent those of their affiliated organizations, or those of the publisher, the editors and the reviewers. Any product that may be evaluated in this article, or claim that may be made by its manufacturer, is not guaranteed or endorsed by the publisher.

## Abbreviations

PWE: Persons with Epilepsy
SDH: Social Determinants of Health.
